# Draft genome sequence of *Enterobacter roggenkampii* strain MBBL_MP5 isolated from dairy cow with clinical mastitis

**DOI:** 10.1128/mra.00106-26

**Published:** 2026-07-20

**Authors:** Kh. Yeashir Arafat, Md. Morshedur Rahman, Md. Anamul Kabir Hemal, Sourav Karmakar, Md. Samiul Islam Shanto, Tofazzal Islam, M. Nazmul Hoque

**Affiliations:** 1 Molecular Biology and Bioinformatics Laboratory, Department of Gynecology, Obstetrics and Reproductive Health, Gazipur Agricultural University 198780, Gazipur, Bangladesh; 2 Institute of Biotechnology and Genetic Engineering (IBGE), Gazipur Agricultural University 198780, Gazipur, Bangladesh; Loyola University Chicago, Chicago, Illinois, USA

**Keywords:** *Enterobacter roggenkampii*, clinical mastitis, dairy cow, antimicrobial resistance, virulence

## Abstract

We report the draft genome of *Enterobacter roggenkampii* MBBL_MP5 isolated from milk of a dairy cow with clinical mastitis. The ~4.7 Mbp genome of MBBL_MP5 encodes 21 antimicrobial resistance genes (ARGs) and 87 virulence factor genes (VFGs), highlighting its possible role in bovine mastitis and relevance to public health.

## ANNOUNCEMENT

Mastitis, an inflammatory condition affecting the mammary glands, poses a significant economic burden on the dairy industry (1–4). It is primarily caused by pathogenic bacteria, although opportunistic microorganisms can also contribute to disease development (5, 6). Among these, *Enterobacter* species, including *E. cloacae* (12.6%), *E. sakazakii* (1.1%) (7), and, more recently, *E. roggenkampii*, have been increasingly associated with bovine mastitis (8, 9). Here, we report the draft genome sequence of *E. roggenkampii* strain MBBL_MP5 isolated from the milk of a dairy cow suffering from clinical mastitis (CM) on a dairy farm in Gazipur (24.09° N, 90.41° E), Bangladesh.

A milk sample (10 mL) was aseptically collected from a lactating cow with CM, as examined by the California Mastitis Test (10–12). Subsequently, 100 µL of the sample was enriched in 5 mL of nutrient broth and incubated overnight at 37°C (13, 14). After enrichment, 2 µL of broth was streaked onto nutrient agar (HiMedia, India) and incubated at 37°C for 24 h. Presumptive single colonies were subcultured onto MacConkey (HiMedia, India) and chromogenic agar (Liofilchem, Italy) for overnight incubation at 37°C. Preliminary identification of *E. roggenkampii* was based on colony morphology (pink on MacConkey, green on chromogenic) and Gram staining (gram-negative rods) (8, 15, 16). Species level identification was performed by using VITEK-2 system v9.01 (17). Genomic DNA was extracted from a pure culture of MBBL_MP5 isolate using QIAamp DNA Mini Kit (QIAGEN, Germany) from the nutrient broth (18, 19). Libraries were prepared with 1 ng DNA using the Nextera DNA Flex Kit (Illumina, USA), and whole-genome sequencing (WGS) was performed using the Illumina MiSeq platform (2 × 250 bp) (20). Raw read quality was evaluated with FastQC v0.11.3 (21), and reads with low quality were subsequently trimmed with Trimmomatic v0.39.2 (22). High-quality reads (Phred score >30) were assembled using SPAdes v4.2.0 (23) and annotated through the National Center for Biotechnology Information (NCBI) Prokaryotic Genome Annotation Pipeline v6.6 (24). Genome completeness was assessed using CheckM v1.2.3 (25). ARGs and VFGs were identified using CARD v3.2.4 (26) and VFDB v6.0 (27), respectively, both integrated into the ABRicate pipeline (https://github.com/tseemann/abricate). CRISPR arrays were detected using CRISPRCasFinder v4.2.20 (28). The distribution of subsystems was determined via the RAST v2.0 server (29), and the potential pathogenic risk to humans was assessed using PathogenFinder v1.1 (30). Default software parameters were applied unless specified otherwise.

**Fig 1:**
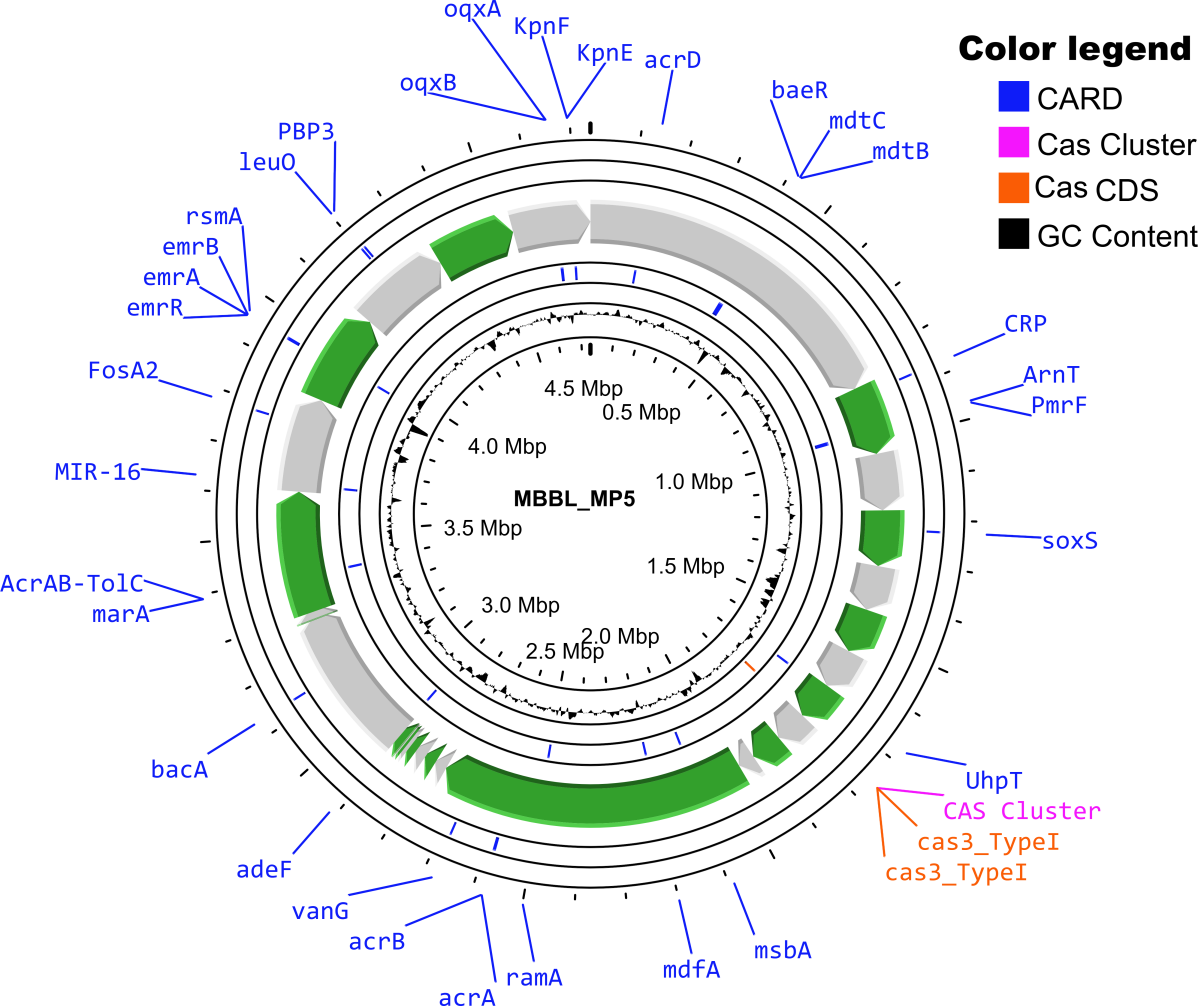
Circular genome map of *E. roggenkampii* strain MBBL_MP5. From the outermost ring toward the center, antibiotic resistance genes (ARGs) are indicated in blue; coding sequences (CDSs) representing CRISPR-associated (Cas) proteins are in orange; and Cas cluster is in purple. GC content is shown as a black plot. The circular genomic map was created using the Proksee server (31).

The genomic characteristics of *E. roggenkampii* MBBL_MP5 are summarized in Table 1 and Fig. 1. The genome is approximately 4.7 Mbp in length, has a guanine-cytosine (GC) content of 56%, an average coverage of 58.35×, and is assembled into 35 contigs. The genome comprises 4,569 genes, including 4,470 coding sequences (CDSs), of which 4,413 are predicted to encode proteins. Moreover, the strain exhibits a very high predicted pathogenicity score of 0.846 (out of 1) and is associated with 48 pathogenic protein families, underscoring its potential relevance to both animal and human health.

**TABLE 1:** Genomic features of *Enterobacter roggenkampii* MBBL_MP5 isolated from milk of a dairy cow diagnosed with clinical mastitis

Genomic features	*E. roggenkampii* MBBL_MP5
GenBank accession	JBSCTX000000000.1
BioSample accession	SAMN53123442
Assembled genome size (bp)	4,739,640
GC (%)	56
Genome completeness (%)	99.48
Genome coverage (×)	58.35×
*N*_50_ value (bp)	243,916
Number of contigs	35
Genes (total)	4,569
CDSs (total)	4,470
Genes (coding)	4,413
CDSs (with protein)	4,413
Genes (RNA)	99
rRNAs	9, 2, 2 (5S, 16S, 23S)
Complete rRNAs	7, 1, 1 (5S, 16S, 23S)
Partial rRNAs	2, 1, 1 (5S, 16S, 23S)
tRNAs	78
ncRNAs	08
Pseudo genes (total)	57
CDSs (without protein)	57
Pseudo genes (ambiguous residues)	0 of 57
Pseudo genes (frameshifted)	23 of 57
Pseudo genes (incomplete)	38 of 57
Pseudo genes (internal stop)	12 of 57
Pseudo genes (multiple problems)	12 of 57
Number of ARGs	21
Number of VFGs	87
Number of subsystems	377
Pathogenicity score	0.846 (out of 1)
Pathogenic families	48
Matched not pathogenic families	11

## Data Availability

The whole-genome project of the *Enterobacter roggenkampii* MBBL_MP5 has been deposited in GenBank and the NCBI Sequence Read Archive (SRA) under BioProject accession number PRJNA1358723 with SRA accession number SRS27016258. The versions described in this paper are version JBSCTX000000000.1.
